# Unraveling the *in vitro* secretome of the phytopathogen *Botrytis cinerea* to understand the interaction with its hosts

**DOI:** 10.3389/fpls.2015.00839

**Published:** 2015-10-09

**Authors:** Raquel González-Fernández, José Valero-Galván, Francisco J. Gómez-Gálvez, Jesús V. Jorrín-Novo

**Affiliations:** ^1^Department of Chemical and Biological Science, Biomedicine Science Institute, Autonomous University of Ciudad JuárezCiudad Juárez, México; ^2^Agroforestry and Plant Biochemistry and Proteomics Research Group, Department of Biochemistry and Molecular Biology, University of Córdoba, Agrifood Campus of International Excellence (ceiA3)Córdoba, Spain

**Keywords:** *Botrytis cinerea*, secretomics, plant pathogenic fungi, fungal secretome, fungi–plant interactions

## Abstract

*Botrytis cinerea* is a necrotrophic fungus with high adaptability to different environments and hosts. It secretes a large number of extracellular proteins, which favor plant tissue penetration and colonization, thus contributing to virulence. Secretomics is a proteomics sub-discipline which study the secreted proteins and their secretion mechanisms, so-called secretome. By using proteomics as experimental approach, many secreted proteins by *B. cinerea* have been identified from *in vitro* experiments, and belonging to different functional categories: (i) cell wall-degrading enzymes such as pectinesterases and endo-polygalacturonases; (ii) proteases involved in host protein degradation such as an aspartic protease; (iii) proteins related to the oxidative burst such as glyoxal oxidase; (iv) proteins which may induce the plant hypersensitive response such as a cerato-platanin domain-containing protein; and (v) proteins related to production and secretion of toxins such as malate dehydrogenase. In this mini-review, we made an overview of the proteomics contribution to the study and knowledge of the *B. cinerea* extracellular secreted proteins based on our current work carried out from *in vitro* experiments, and recent published papers both *in vitro* and *in planta* studies on this fungi. We hypothesize on the putative functions of these secreted proteins, and their connection to the biology of the *B. cinerea* interaction with its hosts.

## Introduction

Phytopathogenic fungi can invade and colonize their plant host to obtain the nutrients because they are able to secrete a set of extracellular proteins and other metabolites (Gonzalez-Fernandez et al., 2010). The secretome has been recently defined as “the global group of secreted proteins into the extracellular space by a cell, tissue, cell, organ, or organism at any given time and conditions through known and unknown secretory mechanisms involving constitutive and regulated secretory organelles” (Agrawal et al., 2013). In the case of phytopatogenic fungi, the secretome consists of pathogenicity and virulence factors, which favor host tissue penetration and colonization in the susceptible plant (Girard et al., 2013). Due to the need to study these secretomes, the term secretomics emerged, and was defined as “the global study of proteins that are secreted by a cell, a tissue or an organism” (Vincent and Bedon, 2013). Many studies have been performed by using both classical approaches and modern–omic techniques through *in planta* experiments to unravel the plant–fungus interaction mechanisms (Allwood et al., 2008; Bhadauria et al., 2009; Tan et al., 2009; Bhadauria et al., 2010; Quirino et al., 2010; Afroz et al., 2011; Dean et al., 2012). As a proteomics sub-discipline, secretomics has contributed significantly to the study of the phytopathogenic fungus secretome by using *in vitro* experiments (González-Fernández and Jorrin-Novo, 2010, 2012; Girard et al., 2013; Vincent and Bedon, 2013). *Botrytis cinerea* Pers. Fr. (teleomorph *Botryotinia fuckeliana* (de Bary) Whetzel) is a necrotrophic pathogen with a wide host range, including pre- and post-harvest plant species, and it causes important economic losses in agriculture (Elad et al., 2007). The *B. cinerea* infection process includes host surface penetration, host tissue killing and primary lesion formation, lesion expansion, tissue maceration, and conidiation (van Kan, 2006). All these stages are mainly achieved by producing secreted proteins and other compounds, including the secretion of cell wall-degrading enzymes (CWDEs), the production of non-specific phytotoxic metabolite (botrydial and botcinolides), the boost of an oxidative burst because of reactive oxygen species (ROS) accumulation, and molecules which induce the plant hypersensitive response (HR; Williamson et al., 2007). In the last years, the use of complementary gel-based and gel-free proteomic approaches has provided important findings in the understanding of *B. cinerea* pathogenicity and virulence in *in vitro* (Gonzalez-Fernandez and Jorrin-Novo, 2012; Gonzalez-Fernandez et al., 2013; González-Fernández et al., 2014) and *in planta* (Shah et al., 2012) experiments.

## The *B. cinerea* secretome unraveled from *In vitro* Proteomic Studies

In fungi, extracellular proteins may be secreted by both the classical pathway, via endoplasmatic reticulum and the Golgi complex, and unconventional export route non-mediated by ER-derived (Girard et al., 2013; Vincent and Bedon, 2013). The *B. cinerea* secretome analysis by using Fungal Secretome Database (FSD; http://fsd.snu.ac.kr/website) showed that 16% of the gene products are predicted to be secreted proteins by the canonical pathway, in which proteins have an N-terminal peptide signal (Choi et al., 2010). This percentage should be increased because it is suggested that various kinds of non-classical export pathways may exist in *B. cinerea* (Jain et al., 2008).

Most studies about *B. cinerea* secretome have been carried out through *in vitro* experiments, mainly because of two problems: (i) the fungal secretome is a complex analysis due to the ratio cell concentration fungus/host, and (ii) the genomic annotation quality for the two partners (Girard et al., 2013; Vincent and Bedon, 2013). To avoid the first difficulty, the *in vitro* experimental protocols try to simulate the *in vivo* conditions, where the fungus is cultured in the presence of more or less-purified fractions of its plant host (Shah et al., 2009a,b; Espino et al., 2010; Fernandez-Acero et al., 2010; González-Fernández et al., 2014). With respect to the second difficulty, it is essential that the fungal and plant genomes be sequenced in order to distinguish between fungal and plant proteins (Girard et al., 2013; Vincent and Bedon, 2013).

In the last years, great efforts have been made to explain the *B. cinerea* secretome complexity and versatility using secretomics from *in vitro* experiments. One of the first studies showed that changes during the fruit ripening process seemed to have an important role in the latent infection activation, which is probably not only dependent on changes in the pectin esterification degree of the plant cell wall (Shah et al., 2009b). By the other hand, this fungus showed significant changes in the composition and relative abundance of secreted proteins that are specific to a particular growth condition (Shah et al., 2009a,b; Espino et al., 2010; Fernandez-Acero et al., 2010; González-Fernández et al., 2014). In the presence of favorable nutrient sources, the fungus increased its protein secretion (Fernandez-Acero et al., 2010). Another key point in the establishment of a successful infection is the extracellular secreted proteins during the spore germination on the plant surface. The early secretome composition was not as variable as it could be expected, and *B. cinerea* seems to secrete a common set of proteins during its germination regardless of growth condition, together with a lower number of proteins that are specifics for these growth conditions (Espino et al., 2010). The aspartic protease BcAP8, a α-amylase 1, and a cerato-platanin domain-containing protein (BcPls1) were detected in the six strains. Two different pectinesterases, one endo-polygalacturonase (PG), a glucan-1,3-β-glucosidase, a glucoamylase, a carboxypeptidase S1, and a choline dehydrogenase were detected in some of the six strains (González-Fernández et al., 2014). Moreover, some hypothetical proteins, which showed differences among strains, were identified in secretome (González-Fernández et al., 2014). An example was the predicted protein BC1G_08642.1, which contains a carbohydrate-recognition domain similar to those ones included in plant and bacterial AB-toxins, glycosidases, or proteases (González-Fernández et al., 2014).

The pH was also shown to affect the *B. cinerea* secretome. Proteins related to proteolysis as the BcAP8, a family S53 protease, a serine-type carboxypeptidase, and a metalloprotease (Merops M35) were induced at pH 4 (similar to mature fruit environment), whereas at pH 6 (similar to leaf environment) most of the up-accumulated proteins were CWDEs (Li et al., 2012). These results were in concordance because ripe fruits generally have lower tissue pH and weakened cell walls, and they accumulate a lot of pathogenesis-related proteins for defense, so inducing protease secretion is more important than the CWDE secretion (Manteau et al., 2003). In contrast, for leaves and stem, which have higher tissue pH and harder cell walls, CWDEs are secreted in greater quantity than the proteases (Manteau et al., 2003). Therefore, pH present in each tissue could regulate the expression of secreted proteins in *B. cinerea* to activate the machinery required for invasion. Finally, metals present in the ambient as cooper, zinc, nickel, or cadmium, also modified the oxidoreductase production and CWDE secretion (Cherrad et al., 2012).

The comparison of these data suggests that *B. cinerea* secretes a common set of proteins as well as a pool of different ones, depending on the *in vitro* growth conditions and the strain, which were used, and making the secretome highly adaptive. Therefore, *B. cinerea* may greatly change the composition of the secreted protein set to satisfy the requirements of these different growing conditions. Next, we are going to discuss the importance of some *B. cinerea* secreted proteins, which have been identified by using a proteomic approach from *in vitro* experiments, and we hypothesize about their putative function in the *B. cinerea* interaction with its host (**Table 1**). All proteins, which are described in **Table 1**, except the pectinesterase BC1G_06840.1, were predicted to be secreted by the classical secretion pathway (Espino et al., 2010; González-Fernández et al., 2014).

**Table 1:** Secreted proteins discussed in this mini-review, which were identified by a proteomic approach from both *in vitro* and *in planta* studies, and which may be involved in the *B. cinerea*–host interaction.

Name	Description	Gene name^a^	SignalP^b^	Reference
**Cell wall-degrading enzymes**				
Pectinesterases	They de-esterify pectins and facilitate the subsequent action of the polygalacturonases (PGs) and pectate lyases.	BC1G_11144.1 BC1G_06840.1 BC1G_00617.1	Yes No Yes	Shah et al., 2009a,b, 2012; Espino et al., 2010; Fernandez-Acero et al., 2010; González-Fernández et al., 2014
Endo-PGs	They hydrolyze the internal (1–4) linkage between D-galacturonic acid units of pectin.	BC1G_11143.1 BC1G_13137.1 BC1G_13367.1 BC1G_04246.1 BC1G_02003.1	Yes Yes Yes Yes Yes	Shah et al., 2009a,b, 2012; Espino et al., 2010; Fernandez-Acero et al., 2010; González-Fernández et al., 2014
Pectin lyases	They cleave polygalacturonic acid into oligogalacturonides.	BC1G_07527.1 BC1G_12017.1 BC1G_12517.1	Yes Yes Yes	Shah et al., 2009a,b, 2012; Espino et al., 2010; Fernandez-Acero et al., 2010; González-Fernández et al., 2014
**Proteases**				
BcAP8	It involves in proteolysis	BC1G_03070.1	Yes	Shah et al., 2009a,b; Espino et al., 2010; Fernandez-Acero et al., 2010; González-Fernández et al., 2014
**Oxidative burst**				
Glyoxal oxidase	It involves in the production of H_2_O_2_	BC1G_01204.1	Yes	Shah et al., 2009b, 2012; Espino et al., 2010; González-Fernández et al., 2014
**Host hypersensitive response (HR)**				
BcPsl1	Elicitor that induces the HR in plants	BC1G_02163.1	Yes	Shah et al., 2009a,b, 2012; Espino et al., 2010; González-Fernández et al., 2014
**Carbohydrate metabolism**				
Glucan β-1,3-glucosidase	It involves in the metabolism of β-1,3-glucans	BC1G_11898.1	Yes	Shah et al., 2009b; Espino et al., 2010; González-Fernández et al., 2014
β-1,3-endoglucanase	It involves in the metabolism of β-1,3-glucans	BC1G_07319.1 BC1G_02551.1 BC1G_00594.1 BC1G_09079.1	Yes Yes Yes Yes	Shah et al., 2009a,b; Espino et al., 2010
Exo-β-1,3-glucanase	It involves in the metabolism of β-1,3-glucans	BC1G_01033.1 BC1G_02731.1	Yes Yes	Shah et al., 2009b; Espino et al., 2010

*^a^Gene name of the different protein isoforms for the B05.10 strain (*Botrytis cinerea* sequencing project, Broad Institute), which were identified through *in vitro* experiments by a proteomic approach.*

*^b^Signal peptide prediction calculated by using the SignalP server (http://www.cbs.dtu.dk/services/SignalP/), indicating the identification (Yes) or not (No) of signal peptide.*

### Proteins Related to Carbohydrate Metabolism

Several enzymes could take part in the metabolism of β-1,3-glucans that are part of the fungal cell wall. This polysaccharide is secreted by *B. cinerea* in high amounts to the medium for which several functions have been proposed, including extracellular energy storage, or the adhesion of the conidia to plant surface during germination (Stahmann et al., 1992). A glucan β-1,3-glucosidase, a β-1,3-endoglucanase, and an exo-β-1,3-glucanase were secreted early in *B. cinerea* development (Espino et al., 2010). The same β-1,3-endoglucanase and other glucanases were detected in the *B. cinerea* interaction with tomato (Shah et al., 2012). The degradation of β-1,3-glucans may contribute to activating the induction of the programmed cell death in plant cells by generating elicitors in the form of β-(1,3)(1,6)-oligomers (Espino et al., 2010).

### Cell Wall-degrading Enzymes

*Botrytis cinerea* is provided with a set of CWDEs able to degrade the cell wall to allow plant tissue colonization to get nutrients (Prins et al., 2000; Shah et al., 2009b; Gonzalez-Fernandez and Jorrin-Novo, 2012). Despite having a broad spectrum of host plant species, this fungus has predilection for dicotyledonous plants with cell walls rich in pectin, which usually is highly methyl-esterified, in order to protect them from fungal PGs and pectate lyases (Prins et al., 2000). Therefore, pectin-degrading enzymes, such as pectinesterases, PGs, and pectate lyases, have a very important role in the cell wall degradation and successful fungal invasion (Kars and van Kan, 2007; Zhang and van Kan, 2013). Recently, the *B. cinerea* BcDW1 genome was sequenced, and the complete genes of a large set of candidate secreted CWDEs were found, among which were found 19 PGs, 15 xyloglucanases, 10 cutinases, and 9 pectin/pectate lyases (Blanco-Ulate et al., 2013). These enzymes are categorized as carbohydrate-active enzymes (CAZymes), which are proteins that degrade, modify, or create glycosidic bonds, and that are also included in diverse glycoside hydrolase (GH) families (Kubicek et al., 2014). By a genome-wide transcriptional profiling analysis, 229 potentially secreted CAZymes were expressed in three different host infected with *B. cinerea* (Blanco-Ulate et al., 2014). These results suggest that *B. cinerea* targets analogous wall polysaccharide matrix on leaves and fruit, and may selectively attack host wall polysaccharide substrates depending on the host tissue.

Two pectinesterases, the endo-PG BcPG1 and the pectin lyase A, were evidenced in the *B. cinerea* secretome by using a combination of 1-DE-MALDI-TOF/TOF MS/MS and label-free shotgun nUPLC–MS^E^ techniques (González-Fernández et al., 2014), as well as in the *B. cinerea* interaction with tomato (Shah et al., 2012). It is known that endo-PGs show not only an elevated genetic variation, and a specialization among them, but also a potential diversification, interacting directly with host defenses (Rowe and Kliebenstein, 2007). Two endo-PGs (BcPG1 and BcPG2) out of the six previously characterized have been reported to be required for full virulence (Choquer et al., 2007; Zhang et al., 2014). For example, the BcPG1 was only detected in five of six wild-type strains (González-Fernández et al., 2014), and in the early secretome (Espino et al., 2010). Moreover, the BcPG1 was secreted by the B05.10 strain grown in glucose, tomato, or kiwifruit, although it was almost absent in strawberry (Espino et al., 2010). This enzyme exemplifies the adaptable secretome nature depending on the strain specificity by its host, and the diversity of the *B. cinerea* armament resulting in an over-kill strategy. Thus, some proteins, which may be required for full virulence to attack one host, may differ from others needed to invade another different host.

### Proteases

A high amount of proteases have been identified in the *B. cinerea* secretome (Shah et al., 2009a; Espino et al., 2010; Fernandez-Acero et al., 2010; González-Fernández et al., 2014). It could be explained due to their role (Espino et al., 2010): (i) generating amino acids to sustain fungal growth; (ii) contributing to cell wall softening, and, therefore, to fungal hyphal penetration; and (iii) degrading the defense proteins which plants secrete against the pathogens. The high diversity of the proteases found may also be reflecting the diverse nature of their substrate. Plant proteins have a great variety of structures by nature, and it may be needed a diverse pool of protease for its degradation (Espino et al., 2010). One of the most abundant proteins secreted by *B. cinerea* is the BcAP8 (Shah et al., 2009a; Espino et al., 2010; Fernandez-Acero et al., 2010; González-Fernández et al., 2014). This protease was detected in the secretome of the six wild-type strains analyzed, varying in abundance depending on the strain (González-Fernández et al., 2014). Besides, its absence in the secretome of B05.10 strain caused the reduction of up to 90% in the secreted protease activity, a rise in the intensity of high molecular weight protein bands in the 1-DE profile, and a decrease, or even the disappearance of the smallest bands (Espino et al., 2010). However, in other study, there was no changes in virulence between the *Bcap8* knockout mutant and the wild-type strain B05.10 in tomato leaves and fruit (ten Have et al., 2010), and the BcAP8 was not detected in the proteomic analysis from *B. cinerea*–tomato interaction (Shah et al., 2012). The result indicated that BcAP8 might not be responsible for virulence in *B. cinerea*. The loss of BcAP8 activity in *ΔBcap8* mutant may be partially compensated by expression of other genes from the *Bcap* family, or BcAP8 activity plays other important roles in the life cycle of the pathogen (Li et al., 2012), supporting the fact of the great adaptability of *B. cinerea*.

### Proteins Involved in Oxidative Burst

Other key mechanism in the *B. cinerea* invasion strategy is the active generation of an oxidative burst by the fungus during the first infection stages (van Kan, 2006; Choquer et al., 2007; Amselem et al., 2011). Several enzymes have been studied as potential ROS generators, such as a Cu–Zn superoxide dismutase (BcSOD1), whose mutation results in reduced virulence in several different hosts, and a glucose oxidase (BcGOD1; Rolke et al., 2004); however, none of them was found by proteomics (Espino et al., 2010; González-Fernández et al., 2014). There are numerous extracellular enzymes that have been previously characterized as potential ROS generators in fungi (Espino et al., 2010). An example is the glyoxal oxidase, which has proved to be an important determinant of cell morphology and virulence in *B. cinerea, Ustilago maydis*, or *Phanerochaete chrysosporium* (Soanes et al., 2008). This enzyme may also produce H_2_O_2_, together with other enzymes as superoxide dismutase (Soanes et al., 2008). Additionally, a quinoprotein glucose dehydrogenase and a cellobiose dehydrogenase were detected in the early secretome that may also generate ROS (Espino et al., 2010). The glyoxal oxidase and the cellobiose dehydrogenase were also detected in the *B. cinerea* interaction with tomato but only in a ripening inhibited mutant tomato (Shah et al., 2012).

### Proteins Involved in the Activation of Plant Defense Response

Pathogens produce molecules which can activate plant defense responses so-called HR (Jones and Dangl, 2006). Unlike biotrophs, necrotrophic fungi as *B. cinerea* takes benefit of the HR, generating dead tissue around the infected area for rapid colonization of their hosts (Govrin and Levine, 2000; Williamson et al., 2007; Hématy et al., 2009). One protein, which may help to induce HR in plants, may be the BcPls1. This protein is a member of the hydrophobin-like cerato-platanin family, which have been reported to be a secreted protein, acting as elicitors, and, in some cases, as pathogenicity factors (Gaderer et al., 2014; Baccelli, 2015). The BcPls1 was found to have high levels of secretion in the B05.10 strain when it was grown *in vitro* by using different media (Shah et al., 2009a,b; Espino et al., 2010), and *in planta B. cinerea*–tomato interaction (Shah et al., 2012), as well as when different wild-type strains were studied (González-Fernández et al., 2014), suggesting that this protein may play an important role in host–pathogen interactions. It was required for full virulence in *B. cinerea*, and induced necrosis in several hosts (Frías et al., 2011). Some HR symptoms were the induction of autofluorescence and ROS, the electrolyte leakage, and the cytoplasm shrinkage (Frías et al., 2011). All these observations may imply that the cerato-platanin proteins are recognized by the immune system of the plant, and this reconnaissance induces with the programmed death of the affected cells (Frías et al., 2011).

### Proteins Associated to the Toxin Secretion

Oxalic acid has been reported as a pathogenicity factor in *B. cinerea* (Lyon et al., 2007), and in the related necrotrophic fungus *Sclerotinia sclerotiorum* (Kim et al., 2008). Its physiological roles in pathogenesis include: (i) the enhancing of PG activity to promote cell wall degradation, (ii) suppression of the plant oxidative burst, (iii) the plant-protection enzymes inhibition, (iv) involvement in the pH signaling, (v) deregulation of stomatal guard cell closure, (vi) induction of apoptosis-like cell death, and (vii) alteration of the cellular redox status in the plant (Amselem et al., 2011). Oxalic acid secretion makes an optimal acidic environment for the pathogenicity factor expression and secretion in *B. cinerea*, such as CWDEs (Manteau et al., 2003), peptidases (ten Have et al., 2010), and for the phytotoxin biosynthesis (Durán-Patrón et al., 2004). Oxaloacetate is the precursor of oxalic acid. The malate dehydrogenase (MDH) catalyzes the reversible conversion of oxaloacetate and malate. A low-gene expression levels of fungal MDH, in combination with the absence of botrydial and dihydrobotrydial secretion, was found in a less virulent *B. cinerea* strain than in the more virulent one (Fernandez-Acero et al., 2007). Fungal MDH was also found in the mycelium proteins of six *B. cinerea* wild-type strains with significant quantitative differences, being higher in the strains isolated from green material (whose pH > 6; González-Fernández et al., 2014). This implies that fungal MDH plays a key role in the biosynthesis of oxalic acid to produce a more appropriate, ecological niche for the fungal pathogenic activities.

## Conclusion and Future Perspectives

The understanding of how the *B. cinerea* secretome affects the interactions between this fungus and its host has become a hard task due to its adaptability at different growth conditions and plant species. *B. cinerea* populations manifest a significant phenotypic variability with respect to their level of aggressiveness, oxidative burst occurring during the infection, and toxin production (Prins et al., 2000; Elad et al., 2007). Considering the results from the different studies, this fungus seems to be efficiently adapted to their different host plants in terms of host preference rather than in a real host specialization (Choquer et al., 2007). Hosts and their parasites are implicated in an evolutionary fighting marked by an adaptation and a counter-adaptation of host defense and pathogen attack mechanisms. Thus, the selective influence perform by plants may affect the virulence factor evolution at population level (Choquer et al., 2007). Certain virulence factors can be important for one strain on one particular host species, but they might be dispensable on other host species, or they might be dispensable for a different strain.

Secretomics has supplied important advances in the identification of extracellular proteins, which are secreted by *B. cinerea*, and may be involved in the interaction with its host that result in a successful infection. So far, only the 10% of the secreted proteins, which were predicted to be involved in the classical export pathway from *B. cinerea* (Choi et al., 2010), has been identified by proteomic approaches (Gonzalez-Fernandez and Jorrin-Novo, 2012; González-Fernández et al., 2014). Due to its high adaptability to different hosts, more specific studies both *in vitro* and *in planta* need to be made for each host to discover the factors involved in the infection mechanisms. Finally, a very important aspect in secretomics is the knowledge of the secretion pathways (Girard et al., 2013; Vincent and Bedon, 2013). Therefore, other point for further studies may be the secretion pathway study, and the cataloging of the proteins according to their secretion mechanisms.

## Conflict of Interest Statement

The authors declare that the research was conducted in the absence of any commercial or financial relationships that could be construed as a potential conflict of interest.
